# Description of a Digital Work-Flow for CBCT-Guided Construction of Micro-Implant Supported Maxillary Skeletal Expander

**DOI:** 10.3390/ma13081815

**Published:** 2020-04-12

**Authors:** Antonino Lo Giudice, Vincenzo Quinzi, Vincenzo Ronsivalle, Stefano Martina, Orazio Bennici, Gaetano Isola

**Affiliations:** 1 Department of General Surgery and Surgical-Medical Specialties, University of Catania, 95124 Via S. Sofia 78, 95123 Catania, Italy; nino.logiudice@gmail.com (A.L.G.); vincenzo.ronsivalle@hotmail.it (V.R.); gaetano.isola@unict.it (G.I.); 2 Department of Life, Health and Environmental Sciences, University of L’Aquila, Piazzale Salvatore Tommasi 1, 67100 Coppito, L’Aquila, Italy; vincenzo.quinzi@univaq.it; 3 Department of Medicine, Surgery and Dentistry “Scuola Medica Salernitana”, University of Salerno, 84081 Baronissi, SA, Italy; step.martina@gmail.com; 4 Private Practice, 95123 Catania, Italy; odontomax@tiscali.it

**Keywords:** digital dentistry, rapid maxillary expansion, miniscrews-assisted maxillary expansion, maxillary skeletal expander, digital orthodontics

## Abstract

The introduction of miniscrew-assisted rapid palatal expansion (MARPE) has widened the boundaries of orthodontic skeletal correction of maxillary transversal deficiency to late adolescence and adult patients. In this respect, Maxillary Skeletal Expander (MSE) is a particular device characterized by the engagement of four miniscrews in the palatal and nasal cortical bone layers. Thus, the availability of sufficient supporting bone and the perforation of both cortical laminas (bi-corticalism) are two mandatory parameters for mini-screw stability, especially when orthopedic forces are used. Virtual planning and construction of MSE based on cone-beam computed tomography (CBCT)-derived stereolithography (.stl) files have been recently described in the literature. In this manuscript we described: (a) a user-friendly digital workflow which can provide a predictable placement of maxillary skeletal expander (MSE) appliance according to the patient’s anatomical characteristics, (b) the construction of a positional template of the MSE that allows lab technician to construct the MSE appliance in a reliable and accurate position, according to the virtual project planned by the orthodontist on the patient CBCT scans. We also described a case report of an adult female patient affected by skeletal transversal maxillary deficiency treated with MSE appliance that was projected according to the described workflow.

## 1. Introduction

Rapid maxillary expansion (RME) represents the standard treatment for the correction of a skeletal transversal maxillary deficiency in growing subjects [1,2,3,4,5]. To maximize the skeletal splitting of the midpalatal suture, heavy forces are transmitted to the maxilla by the anchored teeth, which are hindered from moving due to the hyalinization of the periodontal ligament. However, a pure skeletal opening is not attainable, while unfavorable effects were found in the anchoring teeth and supporting tissue such as root resorption, marginal bone loss, and reduction of buccal bone thickness [6,7,8,9]. The risk of these side effects increases in adult subjects due to the greater compensatory movement of dento-alveolar complex caused by the difficulty in opening the midpalatal suture [1].

**Figure 1:**
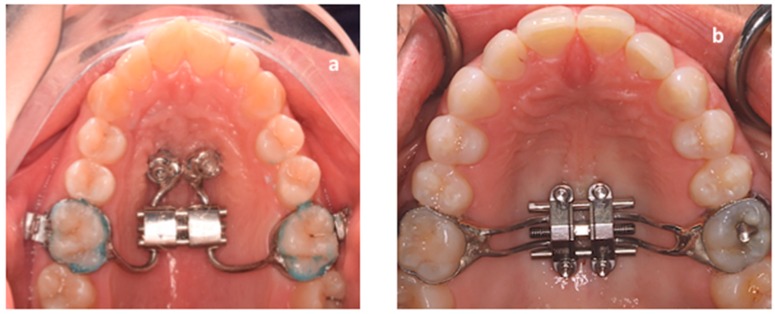
(**a**) Conventional hybrid skeletal anchored expander with bands on first molars and two miniscrews placed in the anterior palate; (**b**) MSE expander placed barely anterior to the soft palate with bands on first molars and four miniscrews inserted via four slots within the expander itself.

In this respect, miniscrew-assisted rapid palatal expansion (MARPE) has been proposed as an effective method to obtain the skeletal opening of the midpalatal suture in late adolescents and adults [10]. This procedure maximizes the expansive forces to the midpalatal suture since the appliance is directly connected to two or four miniscrews inserted into the palatal bone. Usually, two miniscrews are inserted in the anterior palatal region, barely behind the second palatal rugae, then according to this position, the body of the palatal expander is constructed and connected to the miniscrews by retention screws or cementation caps (Figure 1a). By this method, the dental anchorage can be completely avoided (bone-born expander), or the anchorage system can partially rest on dentition (hybrid tooth-born expander) [11,12,13]. Miniscrews are generally placed in the anterior palate, since this region features adequate bone availability at low risk of impacting critical structures [14].

Maxillary Skeletal Expander (MSE, BioMaterials Korea Inc, Seoul, Korea) presents a unique design integrating two molar bands and a body that includes an expansion screw with four slots. Each slot facilitates the placement of the four miniscrews (1.8 mm in diameter and 11 or 13 mm in length). The expander screw is placed between the maxillary first molars, barely anterior to the soft palate (Figure 1b) [15,16,17]. Besides the different locations of miniscrews in the palate, the main difference between the two skeletal anchorage systems is that the MSE expander acts as a rigid insertion guide for the miniscrews placement while in the conventional method the expander is constructed after the placement of the miniscrews. Both procedures seem to be effective in opening the mid-palatal suture in late adolescents and adults [15,16,17]. 

Nevertheless, the morphology of the palate is not uniform, varying from individual to individual [11,12,13,14,15,16,17], and quantitative and qualitative evaluation of bone availability is essential to guarantee good primary stability and reliable anchorage, in particular in terms of parallelism and depth of miniscrew insertion [18,19,20,21,22]. In this respect, cone-beam computed tomography (CBCT) provides three-dimensional quantitative and qualitative assessment of bone structures; furthermore, a digital work-flow based on superimposition of 3D maxillary model on DICOM files has been proposed in order to: (1) virtually insert miniscrews in the palate choosing the most suitable position and angulation and (2) create tailored surgical guides that facilitate the precise directional positioning of palatal miniscrews [22]. 

Concerning the usage of MSE, this appliance is generally planned by using conventional dental stone models and 2D headfilms. However, these diagnostic tools do not allow to accurately define the location of the appliance relatively to midfaceskeletal structures and to assess the the potential risk of affecting critical anatomical areas [15,16,17]. Thus, a digital work-flow would be beneficial to: (1) accurately place the MSE relative to the bizygomatic line, in order to enhance the biomechanics of the expansion, i.e., overcome the resistances of zygomatic buttress bone, (2) maximize the bone thickness at micro-implant insertion sites, (3) define the minimum micro-implant length to penetrate the cortical bone of both palatal vault and nasal floor (bicorticalism), (4) obtain the parallelism between the four microimplants, the midsagittal plane (MP) and the nasal septum.

Cantarella et al. [23] recently documented a digital workflow specifically designed for the MSE appliance. The authors planned the virtual insertion of the MSE (including the four miniscrews) to optimize the bone thickness at miniscrew insertion sites and used specific reference planes, based on CBCT midface skeletal landmarks, to improve the biomechanical effect on the midface. For this purpose, the authors used the CBCT-derived .stl file of the patient, however, this file cannot provide a qualitative assessment of bone availability. In the present paper, we described a digital workflow for the MSE appliance based on CBCT DICOM file, which allows the qualitative discernment between cortical and cancellous bone.

In this respect, the presented method proposes: (1) a preliminary evaluation of quantitative and qualitative bone characteristics, choosing the most suitable location for miniscrews insertion and (2) the design of a fixed positional template in the 3d printed maxillary model, in order to precisely transfer the planned position of the expander to the lab technician for appliance construction. The workflow is illustrated along with a documented case report.

## 2. Materials and Methods 

### 2.1. Patient’s Clinical Characteristics 

#### Clinical Assessment of Palatal Morphology

**Figure 2:**
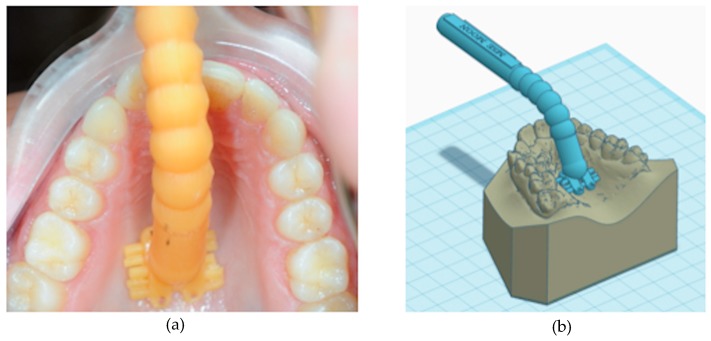
(**a**) Printed template of the expander connected to a handle which facilitates the test of adaptability of the MSE avoiding discomfort to the patient; (**b**) digital version of the 3D template.

Compared to other miniscrew-assisted expanders, the MSE may present some restrictions. In particular, this appliance must be placed in a more posterior position, between the maxillary first molars (barely anterior to the soft palate), in order to concentrate the forces closer to the pterygoid plates that induce great resistance to palatal expansion (Figure 1a,b) [12,23,24]. In addition, the design of the MSE requires a perfect parallelism of the four miniscrews with the nasal septum, with the body of the appliance itself firmly guiding the insertion of the miniscrews. In addition, it is important to select the proper size of MSE since, with skeletal expansion, the MSE must be placed close to the palatal mucos to mitigate the leverage effect on the micro-implants during activations. Thus, a preliminary evaluation of palatal morphology is recommended in order to verify if the patient can be the right candidate for maxillary expansion with this appliance [15,16,17,23]. This evaluation of adaptability of the MSE can be performed during clinical examination by using a 3D printed template of the expander connected to a handle which facilitates the procedure of inspection, avoiding also discomfort to the patient (Figure 2a). 

After scanning the MSE (E3 Scanner, 3Shape A/S, Copenaghen, Denmark), a template of the appliance is digitally designed by using the TINKERCAD software (Version 4.10, AUTODESK, Mill Valley, CA, USA) and 3d printed (Formlabs SLA 3D printer, Somerville, MA, USA). By leaning the template against the palatal vault, it is possible to verify if the MSE can be applied to the patient or there are interferences that prevent a correct placement of the expander. Furthermore, this preliminary evaluation can be performed on patient’s digital models by orienting the .stl file of the MSE according to the shape of the palatal vault (Figure 2b).

### 2.2. Digital Work-Flow

The effectiveness of the maxillary expansion with MSE strictly relies on specific features: parallelism of the four miniscrews with the patient’s nasal septum, bi-cortical skeletal anchorage, posterior position of the expander (barely anterior to the soft palate), absence of excessive pressure on palatal soft tissue. To assure that all these aspects can be satisfied, maxillary cone-beam (CBCT) scans and maxillary .stl file obtained from intra-oral or dental model scans are superimposed in order to identify the most suitable anteroposterior and vertical placement of the appliance based on the width and thickness of the palatal vault. This CBCT-dental model superimposition combines the qualitative and quantitative assessment of palatal bone for the qualitative evaluation of soft tissue characteristics, which allows clinicians to virtually place the miniscrews and the expander according to the previously defined guidelines [12,23,24]. A comprehensive description of the procedure is described below.

### 2.3. CBCT Examination

CBCT examination should be performed with the mouth slightly opened to ensure that the occlusal surfaces do not overlap. A roll of cotton wool can be placed between the patient’s teeth to maintain a stable position during the scan. DICOM file of the image is generated, enabling identification of the anatomical structures in the roof of the palate and, thereby, the most suitable sites for the insertion of the miniscrews-palatal expander complex (see below). In this respect, a careful preliminary evaluation of CBCT scans is mandatory in order to visualize the boundaries of maxillary sinus and the position of posterior dental roots. A small field of view (FOV) is mandatory to avoid unnecessary patient’s exposure to radiations but should be sufficiently wide to identify the anatomical structures in the roof of the palate [25,26,27].

### 2.4. Construction of Positional Template of the MSE

**Figure 3:**
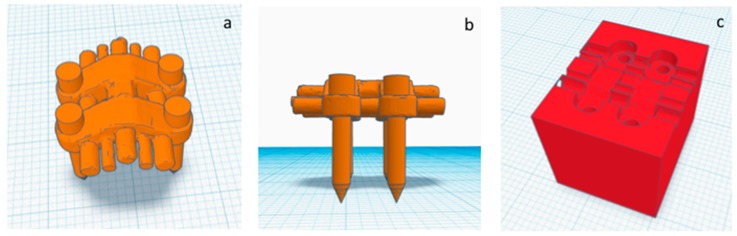
(**a**,**b**) upper and lateral view of the .stl file of the MSE palatal expander with the four miniscrews, (**c**) negative template of the MSE with miniscrews

The construction of the positional template of MSE is performed by using specific functions of the TINKERCAD software (AUTODESK, San Rafael, CA, USA). The original .stl files of the expander screw and the four miniscrews are jointed together by virtually inserting each miniscrew through one of the four slots of the palatal expander. A single .stl file representing the palatal expander with the four miniscrews is created (Figure 3a,b) [23]. A rectangular object (dimension16 × 20 × 16 mm) is created, and the 3d model of the palatal expander with miniscrews is placed inside, then the 3d model was subtracted in order to obtain a negative template of the MSE with miniscrews (Figure 3c).

### 2.5. Virtual Placement of MSE (Negative Template) and Construction of the Final Lab Template

**Figure 4:**
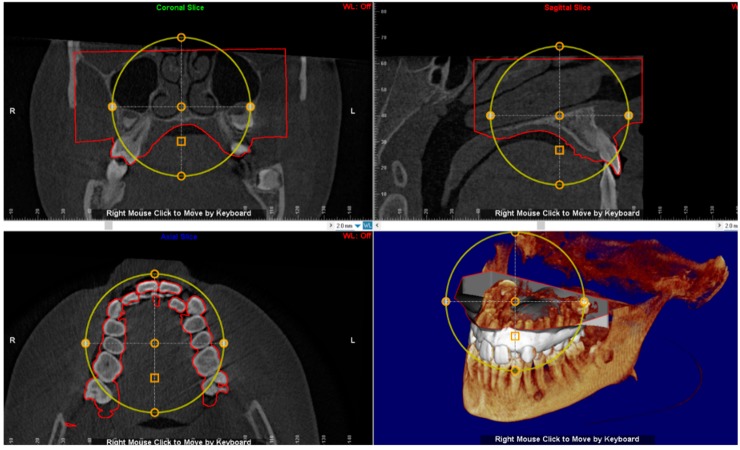
Superimposition of the digital model of maxillary arch onto the DICOM file for identification of the most suitable vertical and anteroposterior placement of the MSE.

**Figure 5:**
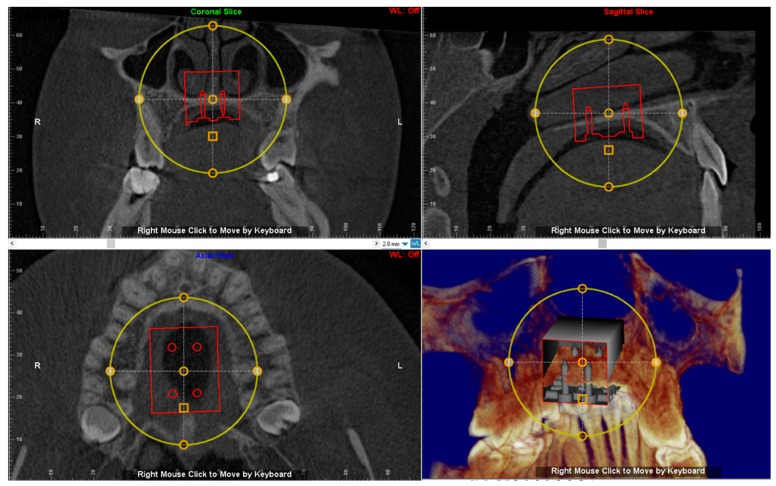
The position of the negative template of MSE is determined in sagittal, coronal and axial views. See the design of the expander with the four miniscrews that are engaged in the cortical bone of the palate and nasal floor.

The digital model of maxillary arch is superimposed onto the CBCT DICOM file by selecting specific landmarks along the dental arch in both files (points-based registration), which allows identification of the most suitable vertical and anteroposterior placement of the MSE according to the following goals: bicortical insertion of the miniscrews, close proximity of the lower base of the MSE to the palatal mucosa and central placement of the screw using the nasal septum as visual reference (Figure 4). All these parameters were evaluated in sagittal, coronal, and axial views, and the position of the negative template of MSE was adjusted according to these parameters (Figure 5).

**Figure 6:**
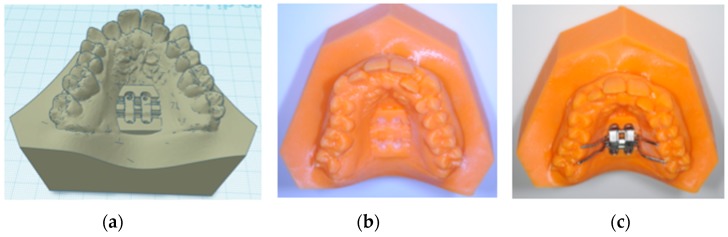
(**a**) .stl file of the negative template of the MSE merged with the maxillary 3d model; (**b**) 3d printed maxillary model includes a template that accurately represents the position of the MSE virtually projected by the clinician; (**c**) this template aid the lab technician in stabilizing the position of the screw during the realization of supporting structures such as the arms connected to anchored teeth.

Then, a single .stl file, including the negative template of the MSE merged with the maxillary 3d model is exported and ready to be manufactured by 3D printing (Figure 6a,b). By this method, the 3D printed maxillary model includes a template that accurately represents the position of the MSE virtually projected by the clinician. Moreover, this template can significantly aid the lab technician in stabilizing the position of the screw during the realization of supporting structures such as the arms connected to anchoring teeth (Figure 6c). Both superimposition, virtual placement of MSE, and export of the merged .stl file are performed by using Dolphin 3D software (version 11.8.06.15 premium; Dolphin Imaging, Chatsworth, CA, USA).

## 3. Case Report

### 3.1. Diagnosis and Treatment Plan

**Figure 7:**
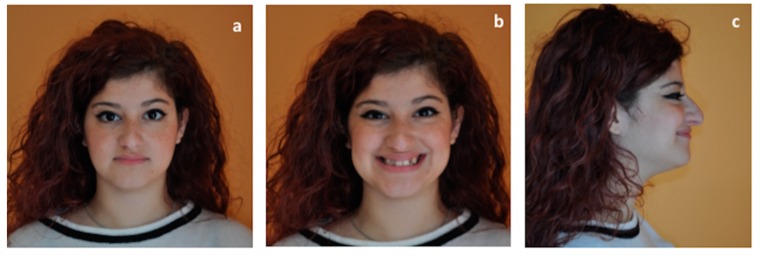
Extra-oral patient’s facial examination. (**a**) frontal view at rest, (**b**) frontal view while smiling, (**c**) right lateral profile at rest.

**Figure 8:**
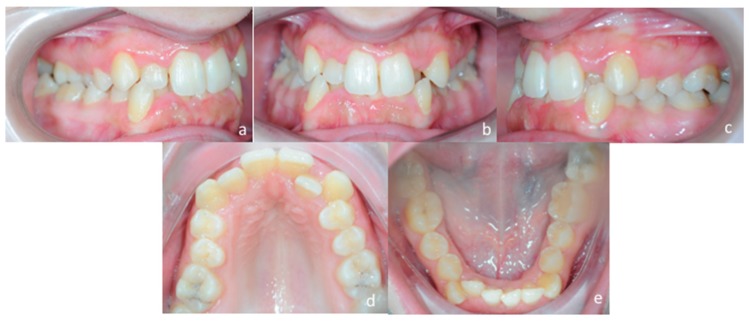
Intra-oral patient’s examination. (**a**) Right lateral occlusion, (**b**) front view, (**c**) left lateral occlusion, (**d**) occlusal view of the maxillary arch, (**e**) occlusal view of the mandibular arch.

**Figure 9:**
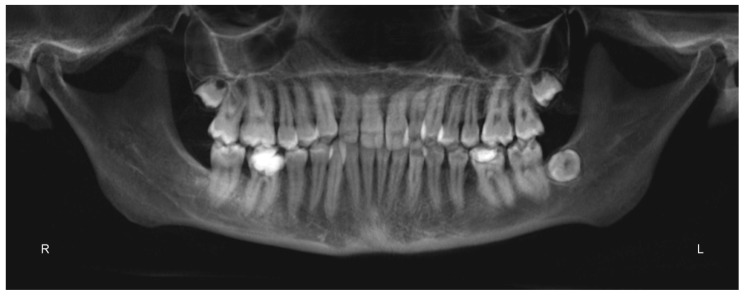
Panorex.

**Figure 10:**
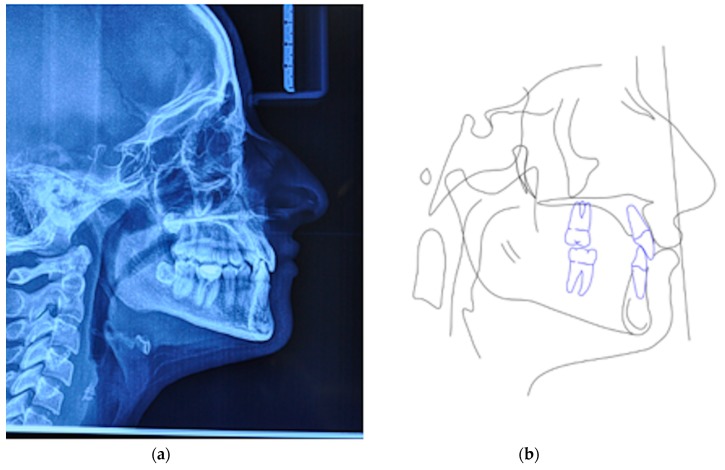
(**a**) Lateral cephalogram, (**b**) pre-treatment cephalometric tracing.

A 25 years old female attended consultation for orthodontic treatment to enhance the aesthetic of the smile. In particular, the chief complain was the crowded teeth along with the presence of wide buccal corridors while smiling. Facial analysis revealed retrognatic profile with labial competence, no gingival exposure during smiling along with extensive buccal corridors (Figure 7a–c). Intra-oral examination revealed class I molar and canine relationships, significant maxillary and mandibular crowding, mild maxillary transversal deficiency with cross-bite on the right side due to mandibular shift toward cross-bite side, significant anterior overbite (Figure 8a–e). Panoramic examination showed healthy condition of the upper first molars that would support the dental anchorage of the MSE device (Figure 9). Cephalometric analysis (Figure 10a,b) confirmed that the patient presented skeletal class I maxillo-mandibular relationship with retruded profile, mesiofacial growth pattern and anterior overbite. Patient approved the usage of photographic and radiographic records for the purpose of publication, by signing a specific form.

The main objective of treatment was to increase arch perimeter in both arches in order to increase space for dental alignment and reduce buccal corridors. Considering the age of the patient, it was decided to expand the maxilla with the aid of skeletal anchorage and we opted for the MSE appliance. 

To ensure that the patient would benefit from therapy with MSE, a preliminary clinical evaluation of palatal morphology was performed, and the handled MSE template was used to verify if this appliance was suitable for the patient (Figure 2a,b).

### 3.2. Digital Workflow for Planning MSE Device

The patient underwent a CBCT examination using the iCAT CBCT Unit (Imaging Sciences International, Hartfield, PA, USA) with the following parameters: 0.3 voxel, 8.9 s, small FOV (8 × 5 cm) at 100 kV and 20 mA. The distance between two slices was 0.3 mm, which provided accuracy in anatomic registration. The intra-oral scan was performed by using Trios 3 and exported in an .stl file using the Orthosystem software (3Shape A/S, Copenaghen, Denmark). Both the DICOM and 3D maxillary model files were imported to Dolphin 3D software (version 11.8.06.15 premium; Dolphin Imaging, Chatsworth, CA, USA) for registration and superimposition.

A negative template of MSE with four miniscrews was created using TINKERCAD software (Version 4.10, AUTODESK, San Rafael, CA, USA), according to the procedure above mentioned (Figure 3a–c). The negative MSE template was imported into Dolphin 3D software to be virtually placed in the palate. In this regard, the position of the negative MSE template was adjusted in order to reach bicorticalism of all the four miniscrews, the close proximity of the lower base of the MSE to the palatal mucosa, central placement of the screw using the nasal septum as a visual reference, the parallelism between miniscrews and nasal septum (Figure 4and Figure 5).

Finally, a single .stl file, including the negative template of the MSE merged with the maxillary 3d model was exported and 3D printed (Figure 6a–c). At this time, the lab technician placed the MSE within the corresponding template on the printed model and designed the final appliance. 

### 3.3. MSE and Miniscrews Insertion

**Figure 11:**
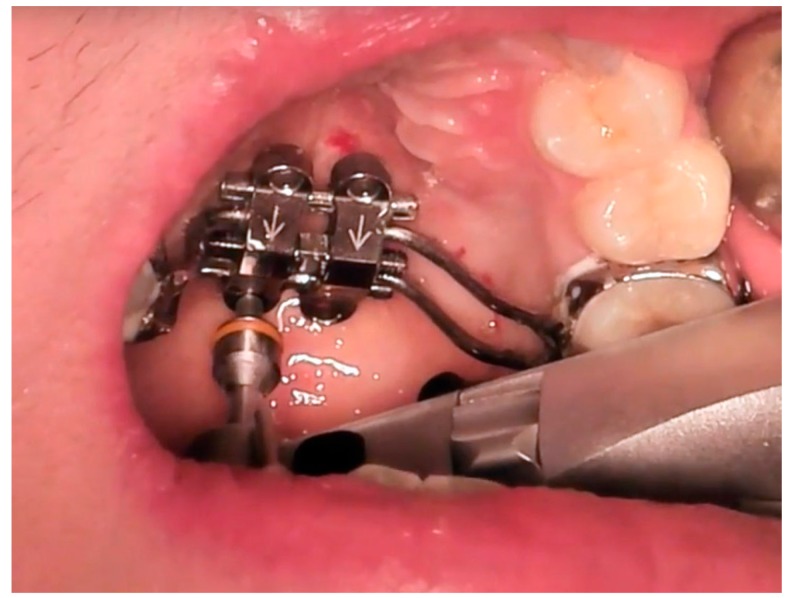
Procedure of miniscrews insertion. Four self-drilling mini-screws (1.8 mm in diameter, 11 mm in length) were used to fix the MSE expander to the palate.

Local anaesthesia to the palatal site (2% articaine) was administered by infiltration in the center of the palate within the area delimitated by first and second molar. Then, MSE appliance was bonded to maxillary first molars using fluoride cement (Ketac Cem, 3M, Saint Paul, MN, USA) and light cured for 30 sec for each anchored tooth. The MSE appliance consisted of four stainless-steel arms soldered to the bands of the first molars and expansion screw with four slots that served as guide for miniscrew insertion (BioMaterials Korea Inc, Seoul, Korea). Four self-drilling mini-screws (1.8 mm in diameter, 11 mm in length) were used to fix the MSE expander to the palate (Figure 11). The device was activated at a rate of two turn per day (0.13 mm widening per turn) until the required expansion was achieved.

### 3.4. Treatment Progress

**Figure 12:**
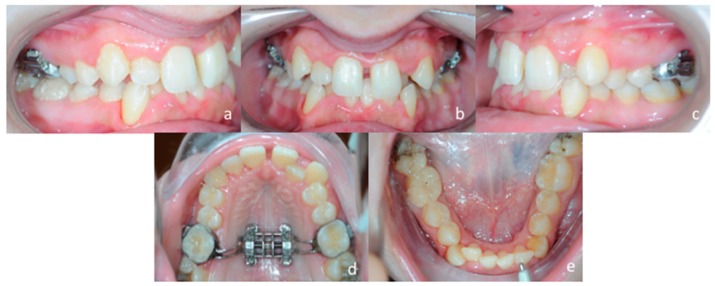
Post expansion intra-oral examination. (**a**) right lateral occlusion, (**b**) front view, (**c**) left lateral occlusion, (**d**) occlusal view of the maxillary arch, (**e**) occlusal view of the mandibular arch.

Evaluation of the effectiveness of the skeletal maxillary expansion procedure was performed two weeks later. Intra-oral inspection showed resolution of the posterior cross-bite at the right side and the appearance of the diastema between maxillary central incisors, which confirmed a skeletal opening of the midpalatal suture (Figure 12a–e). Then, activations were interrupted, and the MSE expander was locked. The patient was seen every month to monitor the retention phase of palatal expansion before bonding fixed orthodontic appliances. 

## 4. Discussion

The use of skeletal anchorage is getting widespread among orthodontists since it facilitates the management of complex orthodontic biomechanics; as a consequence, skeletal anchorage can be useful in treating borderline cases such as transversal maxillary deficiency in adults. In this respect, miniscrews-assisted rapid maxillary expansion was found to effectively open the mid-palatal suture in adults without the necessity to undergo orthognathic surgery [12,24,28,29,30,31,32,33,34,35,36,37].

The effectiveness of skeletal anchorage, however, relies on specific characteristics that can affect primary and secondary stability of the miniscrew [30]. The availability of sufficient supporting bone and the perforation of both cortical laminas (bi-corticalism) are two mandatory parameters for mini-screw stability, especially when orthopedic forces are used, as during skeletal maxillary expansion. In this respect, a quantitative and qualitative evaluation of bone characteristics [30,31,32,33,34,37,38,39,40], as well as the assessment of the most suitable position of the miniscrews in the palate, are critical for the success of maxillary expansion assisted by skeletal anchorage. In this paper, we followed the recent guidelines for digital workflow planning proposed by Cantarella et al. [23] for the MSE appliance, however, we utilized the patient CBCT DICOM file that allows discriminating between cortical and cancellous bone. The present user-friendly digital workflow can provide:

(1) predictable placement of the MSE appliance, according to the patient’s anatomical characteristics
(2) the construction of a negative positional template of the MSE that allows lab technicians to construct the device in a reliable and accurate position, according to the virtual project planned by the orthodontist.

## 5. Conclusions

We described a full digital work-flow (CAD-CAM) that can help clinicians in defining the appropriate placement of micro-implant supported maxillary skeletal expander (MSE) according to quantitative and qualitative bone characteristics of the palate region. This workflow can also enhanced the communication between the orthodontists and lab technicians for the construction of the MSE since it provides a physical negative template for the accurate placement of the screw within the dental cast.
